# Stochastic nonlinear dynamics pattern formation and growth models

**DOI:** 10.1186/1753-4631-1-4

**Published:** 2007-07-05

**Authors:** Leonid P Yaroslavsky

**Affiliations:** 1 Department of Interdisciplinary Studies, Faculty of Engineering, University of Tel Aviv, Tel Aviv 69978, Israel

## Abstract

Stochastic evolutionary growth and pattern formation models are treated in a unified way in terms of algorithmic models of nonlinear dynamic systems with feedback built of a standard set of signal processing units. A number of concrete models is described and illustrated by numerous examples of artificially generated patterns that closely imitate wide variety of patterns found in the nature.

## Background

Problems of pattern formation and growth of forms belong to the most fundamental problems in theoretical biology and other natural sciences [1-4]. In this paper, we treat these problems from the nonlinear dynamics and system theory perspective. Specifically, we regard pattern formation and growth models as versions of pseudo-random number generators and show that they can be described and generated in terms of nonlinear systems with feedback built of a standard set of signal processing units. We show also that quite simple algorithmic models are capable of generating a wide variety of patterns, which closely remind patterns frequently found in the nature such as dendrite patters, labyrinth and zebra skin patterns, papillary patterns, fingerprints and alike. We believe that this approach facilitates unification, quantification and comparison of the growth and pattern formation models and secures their efficient computational implementation.

The paper is organized as following. In Section 2, commonly used generators of pseudo-random numbers are described, represented in terms of the nonlinear dynamic systems with feedback and generalized on this base. In Section 3, it is shown that simple and straightforward modifications of these random number generators give rise to a wide family of stochastic growth models that are illustrated by Eden's type models [5-8] and by several modifications of evolutionary models that originate from Conway's "Game of Life" [8-11]. Section 4 is devoted to an extension of the approach to formation of 2-D stochastic patterns commonly called "texture" images. It suggests regular methods for generating texture images and provides a number of concrete examples of texture generating algorithmic models of different complexity capable, in particular, of imitating quite complex natural textures.

## Pseudo-random number generators

Nothing in Nature is random. A thing appears random only through the incompleteness of our knowledge (B. Spinoza [12])

Anyone who considers arithmetical methods of producing random digits is, of course, in the state of sin. (J. Von Neuman, [12])

In this section, we describe numerical generators of "pseudo-random" numbers that are commonly used in Monte Carlo simulations and show how can they be represented in a form of nonlinear dynamical (evolutionary) systems with feedback that we further, in sections that follow, extend to more sophisticated growth and pattern formation models.

First generators of pseudo-random numbers were suggested by John Von Neuman at the very beginning of the computer era. Since then, many attempts have been undertaken to improve "randomness" of the generated numbers including even attempts to introduce hardware random number generators that exploit "random" nature phenomena such as radioactivity or Brownian motion. Finally, the concept of pseudo-random numbers won overwhelming recognition, and software pseudo-random number generators have become commonly accepted for generating stochastic numbers that seem "random" in particular applications.

These generators produce pseudo-random numbers recursively from one initial number ("seed" number) by using quite simple computational rules. For instance, Knuth [13] recommends an algorithm that can be described by the following recursive relationship

ξ(t)=[C1ξ(t−1)+C2]modC3
 MathType@MTEF@5@5@+=feaafiart1ev1aaatCvAUfKttLearuWrP9MDH5MBPbIqV92AaeXatLxBI9gBaebbnrfifHhDYfgasaacH8akY=wiFfYdH8Gipec8Eeeu0xXdbba9frFj0=OqFfea0dXdd9vqai=hGuQ8kuc9pgc9s8qqaq=dirpe0xb9q8qiLsFr0=vr0=vr0dc8meaabaqaciaacaGaaeqabaqabeGadaaakeaaiiWacqWF+oaEdaahaaWcbeqaamaabmaabaacbmGae4hDaqhacaGLOaGaayzkaaaaaOGaeyypa0ZaamWaaeaacqGFdbWqdaWgaaWcbaacbeGae0xmaedabeaakiab=57a4naaCaaaleqabaWaaeWaaeaacqGF0baDcqGHsislcqqFXaqmaiaawIcacaGLPaaaaaGccqGHRaWkcqGFdbWqdaWgaaWcbaGae0NmaidabeaaaOGaay5waiaaw2faamaaBaaaleaacqqFTbqBcqqFVbWBcqqFKbazcqGFdbWqdaWgaaadbaGae03mamdabeaaaSqabaaaaa@46FB@

Here ***ξ*^(*t*) ^**is a pseudo-random number generated at ***t***-th iteration, ***C*_1_**, ***C*_2_**, and ***C*_3 _**are certain constants, **[·]_mod *C *_**is an operation of finding residual of division of the input value by ***C*_3_**.

**Figure 1:**
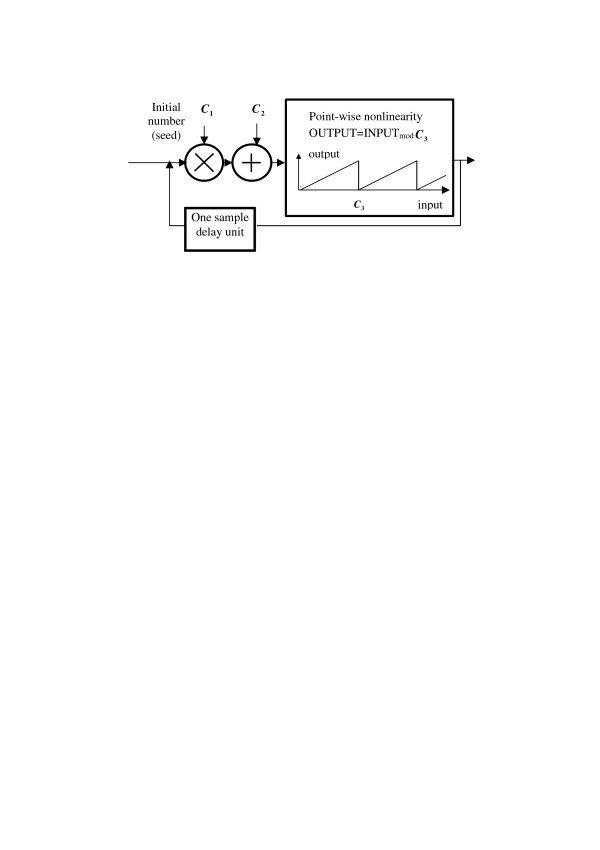
Schematic diagram of a pseudo-random number generator. The graph shows transfer function of the point-wise nonlinearity unit.

This commonly used algorithm generates, one by one, pseudo-random numbers with uniform distribution density in the range [0,1]. The algorithm can be represented by a schematic diagram is shown in Figure 1. Represented in this way, the algorithm is built of the following signal processing units: a multiplication unit, a summation unit, a point-wise nonlinearity unit that implements operation **[·]_mod *C *_**(its transfer function is shown in the box in Figure 1), and a one-sample delay unit. The latter is a very important component of the scheme that makes it recursive, or, in another word, evolutionary.

This scheme represents an example of a very simple nonlinear dynamic (evolutionary) system. It is well known that such systems potentially are prone to cycles and "fixed points", states that, when reached, do not change in the process of iterations (system evolution). A natural requirement to the pseudo-random number generators is that they should avoid cycles and fixed points and provide numbers with nearly uniform distribution and without noticeable correlations. In practice it is achieved by a careful selection of the model parameters ***C*_1_**, ***C*_2_**, and ***C*_3 _**[14].

**Figure 2:**
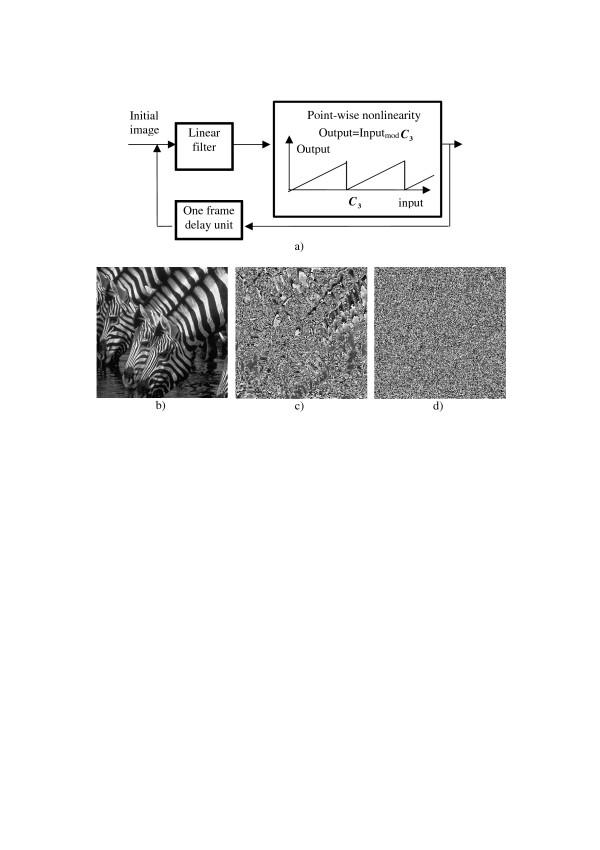
A modification of the pseudo-random number generator with a linear filter in the feedback (a) and examples of an initial image (b) and generated images after one (c) and 10 (d) iterations.

The above scheme can, in a very natural way, be extended to the one presented in Figure 2.

Multiplication and summation units in the scheme of Figure 1 are replaced here by a linear filter, a device that computes output signal by weighted summation of certain number of input samples, the weights being defined by the filter impulse response (point spread function). In addition, one-sample delay unit of the scheme in Figure 1 is replaced by a one-frame delay unit, where frame is a certain group of samples.

If signal samples in this scheme are arranged in a form of a 2D array, they can be displayed as an image. Figure 2b) illustrates an example of evolution in such a system of a natural image taken as a "seed". The linear filter in this example is a simple two-dimensional "box" filter with a uniform 3 × 3 samples impulse response. Such a filter computes, for each image sample (*pixel*), image local mean value over the window of 3 × 3 pixels centered at this sample. A constant ***C*_3 _**in the point-wise nonlinearity was set equal to the half of the image maximal gray level. One can see on this image how the nonlinearity and feedback destroy, in only a few iterations, all pixel correlations that existed in the initial image and generate a 2D array of numbers with no visual correlations.

In what follows, we will use such units, which we call "primary random number generators", as primary units in the stochastic growth and pattern formation models. They will generate inputs to the models and, in addition, they will determine "clocks" of the model evolution.

## Stochastic growth models

In this section, we describe several classical numerical stochastic growth models to show that they can be considered as extensions of above presented pseudo-random number generators and described in terms of nonlinear dynamic system composed of standard signal processing units.

### Eden's type growth models

Stochastic growth models aimed at simulating biological grows have been studied since very first years when digital computers became available [15]. One of the first models was suggested by M. Eden [5,6]. In Eden's model, growth was simulated as a sequence of random "births" taking place on a rectangular lattice with the probability proportional to the number of already "live" cells in the nearest spatial 3 × 3 vicinity of the given cell (left and right neighbors at the same row, three neighbors on the rows from above and three from bottom). Eden's model can be mathematically represented as an recursive equation:

output(k,l)(t)=2Drandb(S8(t−1)/8)⊕output(k,l)(t−1)
 MathType@MTEF@5@5@+=feaafiart1ev1aaatCvAUfKttLearuWrP9MDH5MBPbIqV92AaeXatLxBI9gBaebbnrfifHhDYfgasaacH8akY=wiFfYdH8Gipec8Eeeu0xXdbba9frFj0=OqFfea0dXdd9vqai=hGuQ8kuc9pgc9s8qqaq=dirpe0xb9q8qiLsFr0=vr0=vr0dc8meaabaqaciaacaGaaeqabaqabeGadaaakeaaieWacqWFVbWBcqWF1bqDcqWF0baDcqWFWbaCcqWF1bqDcqWF0baDdaqadaqaaiab=TgaRjabcYcaSiab=XgaSbGaayjkaiaawMcaamaaCaaaleqabaWaaeWaaeaacqWF0baDaiaawIcacaGLPaaaaaGccqGH9aqpcqWHYaGmcqWHebarcqWHYbGCcqWHHbqycqWHUbGBcqWHKbazcqWHIbGydaqadaqaaiab=nfatnaaDaaaleaaieqacqGF4aaoaeaadaqadaqaaiab=rha0jabgkHiTiab+fdaXaGaayjkaiaawMcaaaaakiabc+caViab+Hda4aGaayjkaiaawMcaaGGabiab9vPiflab=9gaVjab=vha1jab=rha0jab=bhaWjab=vha1jab=rha0naabmaabaGae83AaSMaeiilaWIae8hBaWgacaGLOaGaayzkaaWaaWbaaSqabeaadaqadaqaaiab=rha0jabgkHiTiab+fdaXaGaayjkaiaawMcaaaaaaaa@668E@

where **(*k*, *l*) **are pixel coordinates on the lattice, S8t−1
 MathType@MTEF@5@5@+=feaafiart1ev1aaatCvAUfKttLearuWrP9MDH5MBPbIqV92AaeXatLxBI9gBaebbnrfifHhDYfgasaacH8akY=wiFfYdH8Gipec8Eeeu0xXdbba9frFj0=OqFfea0dXdd9vqai=hGuQ8kuc9pgc9s8qqaq=dirpe0xb9q8qiLsFr0=vr0=vr0dc8meaabaqaciaacaGaaeqabaqabeGadaaakeaaieWacqWFtbWudaqhaaWcbaacbeGae4hoaGdabaGae8hDaqNaeyOeI0Iae4xmaedaaaaa@324E@ is the sum of pixel values in 8 neighbor points in the 3 × 3 neighborhood of the given pixel, ***t ***is the iteration index, **2Drandb(*P*) **is a binary 2D array of pseudo-random numbers that take value one with probability ***P ***and ⊕ denotes modulo 2 addition of binary numbers.

**Figure 3:**
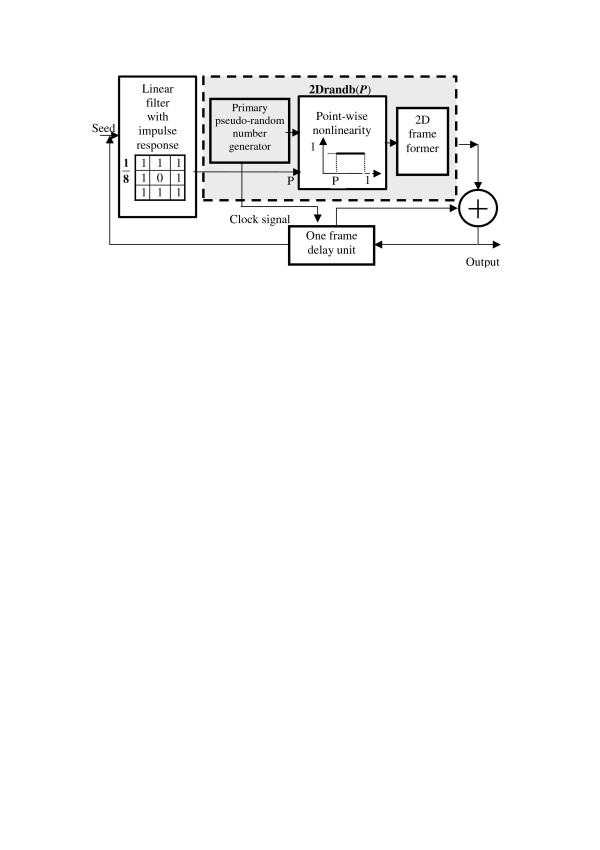
Schematic diagram of the Eden's model. The table in the box "Linear filter" presents the linear filter impulse response. The graph in the box "Point-wise nonlinearity" in shows the nonlinearity transfer function.

Figure 3 shows how this growth model can be implemented in a system that is just a slightly modified and extended version of the system of Figure 2. This system contains, as an individual unit, the "primary" pseudo-random number generator of Figure 1, which is now included in the loop with a linear filter, point-wise nonlinearity, 2D frame former (a unit that converts sequences of numbers into a 2D arrays of numbers), and a one frame delay unit. Impulse response of the linear filter and transfer function of the point-wise nonlinearity are shown in the corresponding boxes in Figure 3. This unit also generates a clock signal for the one-frame delay unit that defines the evolution clock rate.

We assume that the 'primary" pseudo-random number generator generates real numbers in the range [0-1]. The combination of the 'primary" pseudo-random number generator and the point-wise nonlinearity with a threshold transfer function forms the unit **2Drandb(*P*)**, which implements an operation of generating, out of the primary pseudo-random numbers, binary numbers zeros and ones with a given probability ***P ***of ones. On such an array of binary numbers, the linear filter with impulse response as shown in Figure 3 computes the number of ones in the 3 × 3 neighborhood (8-neighbor sum ***S*_8_**) of each pixel thus defining the threshold level of the point-wise nonlinearity.

**Figure 4:**
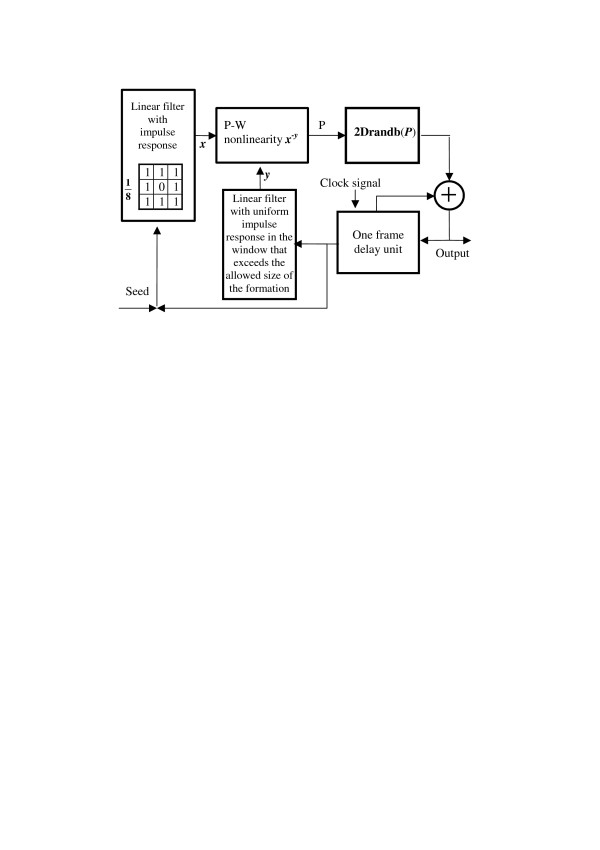
Schematic diagram of the Eden's model with saturation.

Clearly, this simple model describes unlimited growth. One can, however, easily modify this model to simulate drain of "sources of food" by measuring the size of the growing formation and introducing a corresponding saturation to the probability of "birth" as it is shown in schematic diagram of Figure 4.

In this scheme, the **2Drandb(*P*) **unit of Figure 3a is preceded by a **(*x*^-*y*^) **– point-wise nonlinearity that implements the saturation. This modified model can be described by equation

output(k,l)t=randb((S8(t−1))−cSgl(t−1)/8)+output(k,l)t−1,
 MathType@MTEF@5@5@+=feaafiart1ev1aaatCvAUfKttLearuWrP9MDH5MBPbIqV92AaeXatLxBI9gBaebbnrfifHhDYfgasaacH8akY=wiFfYdH8Gipec8Eeeu0xXdbba9frFj0=OqFfea0dXdd9vqai=hGuQ8kuc9pgc9s8qqaq=dirpe0xb9q8qiLsFr0=vr0=vr0dc8meaabaqaciaacaGaaeqabaqabeGadaaakeaaieWacqWFVbWBcqWF1bqDcqWF0baDcqWFWbaCcqWF1bqDcqWF0baDdaqadaqaaiab=TgaRjabcYcaSiab=XgaSbGaayjkaiaawMcaamaaCaaaleqabaGae8hDaqhaaOGaeyypa0JaeCOCaiNaeCyyaeMaeCOBa4MaeCizaqMaeCOyai2aaeWaaeaadaqadaqaaiab=nfatnaaDaaameaaieqacqGF4aaoaSqaamaabmaabaGae8hDaqNaeyOeI0Iae4xmaedacaGLOaGaayzkaaaaaaGccaGLOaGaayzkaaWaaWbaaSqabeaacqGHsislcqWFJbWycqWFtbWudaqhaaadbaGae83zaCMae8hBaWgabaWaaeWaaeaacqWF0baDcqGHsislcqGFXaqmaiaawIcacaGLPaaaaaaaaOGaei4la8Iae4hoaGdacaGLOaGaayzkaaGaey4kaSIae83Ba8Mae8xDauNae8hDaqNae8hCaaNae8xDauNae8hDaq3aaeWaaeaacqWFRbWAcqGGSaalcqWFSbaBaiaawIcacaGLPaaadaahaaWcbeqaaiab=rha0jabgkHiTiab+fdaXaaakiabcYcaSaaa@6E0E@

where Sgl(t−1)
 MathType@MTEF@5@5@+=feaafiart1ev1aaatCvAUfKttLearuWrP9MDH5MBPbIqV92AaeXatLxBI9gBaebbnrfifHhDYfgasaacH8akY=wiFfYdH8Gipec8Eeeu0xXdbba9frFj0=OqFfea0dXdd9vqai=hGuQ8kuc9pgc9s8qqaq=dirpe0xb9q8qiLsFr0=vr0=vr0dc8meaabaqaciaacaGaaeqabaqabeGadaaakeaaieWacqWFtbWudaqhaaWcbaGae83zaCMae8hBaWgabaWaaeWaaeaacqWF0baDcqGHsislieqacqGFXaqmaiaawIcacaGLPaaaaaaaaa@3593@ is a "global" sum over the entire field of growth. It defines the size of the formation on (***t***-1)-th iteration (evolution) step.

**Figure 5:**
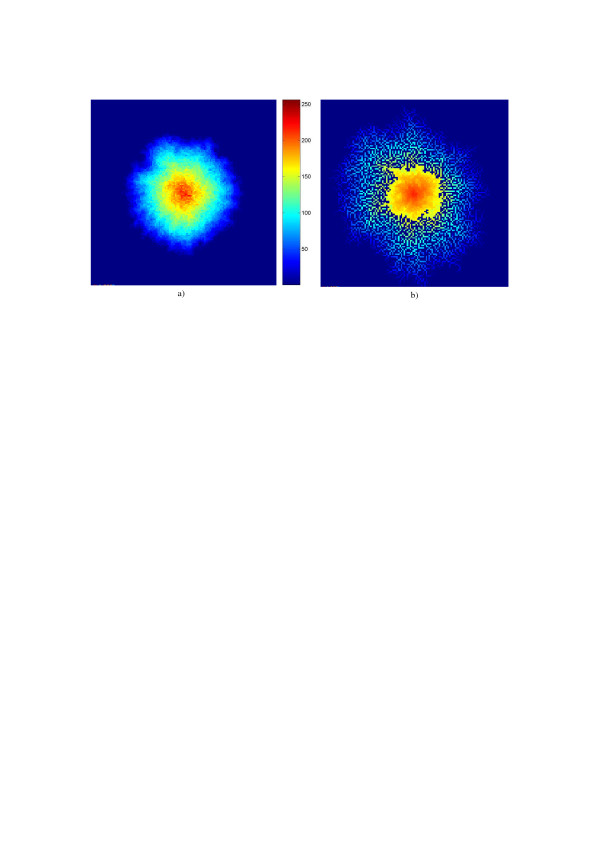
Examples of images generated by the Eden's model with saturation (a) and its modification that evolves into growing dendrites (b). Images are color coded according to the color bar to indicate the "age" of different parts of the patterns.

If saturation is introduced to all probabilities but to the probability of "birth" from only one neighboring live cell, one arrives at a modification of the model, which begins to grow dendrites after (statistically) the cell reaches a certain size. Figs. 5a) and 5b) illustrate the work of these models. Images are displayed here in color that corresponds to the "age", from red to blue, of each pixel (number of evolution steps from its birth). Other modifications of the model aimed, for instance, at imitating dependence of growth from "age" of cells are also more or less straightforward.

### Conway's "Game of Life" and its modifications

A famous mathematical model known as Conway's "Game of Life" [8] represents yet another type of growth models, where cells on a rectangular lattice (raster) can give a "births" or "die out" depending on the number of "alive" and empty ("dead") cells in their nearest spatial neighborhood. The rules of the original "Game of life" are very simple: (i) if an empty cell has exactly 3 "alive" neighbor cells in its 3 × 3 neighborhood in the rectangular lattice, birth takes place in this cell on the next step of the evolution; (ii) if an "alive" cell has less than 2 and more than 3 "alive" cells in the neighborhood it will die on the next step; (iii) otherwise nothing happens. These rules can be formally described by the equation:

output(k,l)t=[output(k,l)t−1]δ(S8t−1−2)+δ(S8t−1−3)
 MathType@MTEF@5@5@+=feaafiart1ev1aaatCvAUfKttLearuWrP9MDH5MBPbIqV92AaeXatLxBI9gBaebbnrfifHhDYfgasaacH8akY=wiFfYdH8Gipec8Eeeu0xXdbba9frFj0=OqFfea0dXdd9vqai=hGuQ8kuc9pgc9s8qqaq=dirpe0xb9q8qiLsFr0=vr0=vr0dc8meaabaqaciaacaGaaeqabaqabeGadaaakeaaieWacqWFVbWBcqWF1bqDcqWF0baDcqWFWbaCcqWF1bqDcqWF0baDdaqadaqaaiab=TgaRjabcYcaSiab=XgaSbGaayjkaiaawMcaamaaCaaaleqabaGae8hDaqhaaOGaeyypa0ZaamWaaeaacqWFVbWBcqWF1bqDcqWF0baDcqWFWbaCcqWF1bqDcqWF0baDdaqadaqaaiab=TgaRjabcYcaSiab=XgaSbGaayjkaiaawMcaamaaCaaaleqabaGae8hDaqNaeyOeI0ccbeGae4xmaedaaaGccaGLBbGaayzxaaaccmGae0hTdq2aaeWaaeaacqWFtbWudaqhaaWcbaGae4hoaGdabaGae8hDaqNaeyOeI0Iae4xmaedaaOGaeyOeI0Iae4NmaidacaGLOaGaayzkaaGaey4kaSIae0hTdq2aaeWaaeaacqWFtbWudaqhaaWcbaGae4hoaGdabaGae8hDaqNaeyOeI0Iae4xmaedaaOGaeyOeI0Iae43mamdacaGLOaGaayzkaaaaaa@662C@

where "alive" and "empty" cells are represented by "ones" and "zeros", respectively, ***δ*(·) **is the Kronecker delta (***δ*(0) **= **1; *δ*(*x *≠ 0) = 0**), S8t−1
 MathType@MTEF@5@5@+=feaafiart1ev1aaatCvAUfKttLearuWrP9MDH5MBPbIqV92AaeXatLxBI9gBaebbnrfifHhDYfgasaacH8akY=wiFfYdH8Gipec8Eeeu0xXdbba9frFj0=OqFfea0dXdd9vqai=hGuQ8kuc9pgc9s8qqaq=dirpe0xb9q8qiLsFr0=vr0=vr0dc8meaabaqaciaacaGaaeqabaqabeGadaaakeaaieWacqWFtbWudaqhaaWcbaacbeGae4hoaGdabaGae8hDaqNaeyOeI0Iae4xmaedaaaaa@324E@ is the sum of the values in 8-neighborhood of (***k*, *l***)-th cell on a rectangular lattice, and ***t ***is the iteration number.

**Figure 6:**
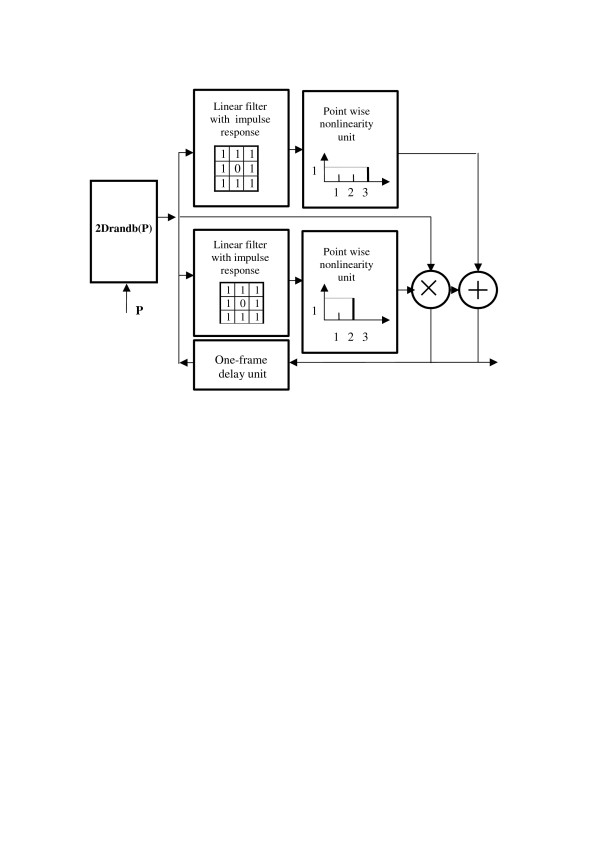
Schematic diagram of the stochastic Conway's "Game of Life" model. Tables and graphs in boxes show impulse response and transfer functions of the corresponding units.

In the original model[8], a deterministic initial distribution of zeros and ones in the field was assumed. By introducing "random" initial distribution of "alive" and empty cells, the model can be made stochastic [9,10]. The corresponding schematic diagram of this model is shown in Figure 6.

As one can see, this diagram contains essentially the same units as the Eden's model, but here they as arranged in 2 parallel branches (one for "births" and one for "deaths"), and the **2Drandb(*P*) **generator of the Eden's model is placed at the input of the model and is used for generating only initial "random" distribution of 1's and 0's for "alive" and empty cells. The evolution clock rate of the model is determined in this model by the one-frame delay unit.

It is well known that the model generates several types of formations:

- Stable formations that once appeared keep staying unchanged unless they are destroyed by other formations;

- Growing crystal-like formations that grow until their fragments form stable formations or die out;

- Cyclic, in course of iterations, formation that repeat themselves with a certain period;

- "Moving", in course of iterations, formations also featuring iteration-wise cycles ("gliders").

**Figure 7:**
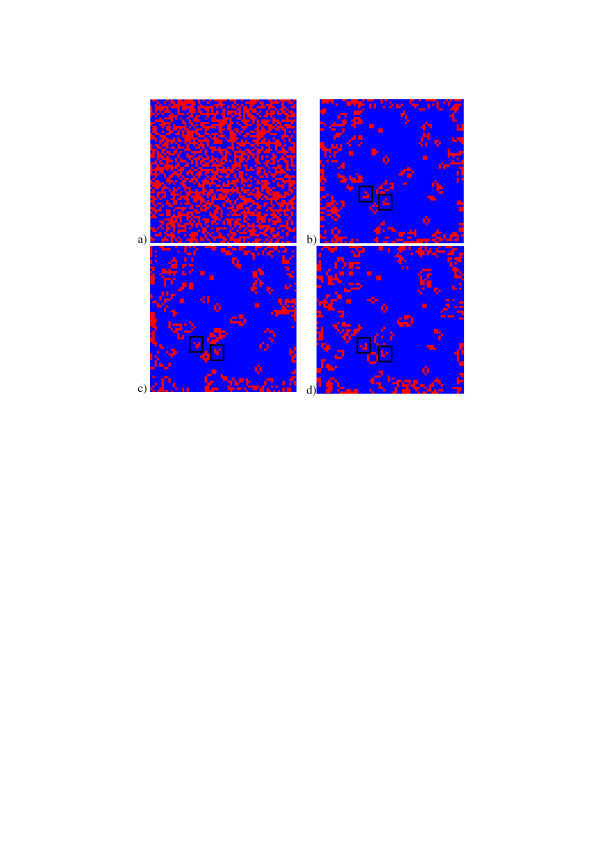
Evolution of pattern a) in the Game of Life model: a) – initial pattern, b) – d) – patterns on 75, 76, and 77-th iterations, correspondingly.

Boundary conditions of the model are important for its evolution. Under pseudo-random boundary conditions, when pseudo-random binary numbers are permanently generated at the borders of the field, the model generates patterns that do not converge to a fixed (stable) ones though always contain certain number of formations that "live" during considerably large number of iterations (evolution steps). Such pattern evolution is illustrated in Figure 7.

One can, for instance, see on these images "randomly" located stable formations such as 2 × 2 pixel square blocks, hexagonal formations called beehives, formations that grow like crystals, and moving formations, or "gliders" (marked in the figures by black boxes), which move across the lattice with a period of 4 evolution steps.

An important parameter of the model is the direction of the spatial interaction. It is defined by the linear filter impulse response. In the original Conway's model, the spatial interaction is almost isotropic: all cell's 8 neighbors play the same role in the defining next state of the cell on each iteration step. In the model of Figure 6, this is reflected in the linear filter isotropic impulse response equal to 1 for all 8 neighbor pixels. In general, the filter impulse response may not be isotropic. In particular, it may define only one-dimensional interaction (only left and right neighbors of each cell affect its next state) thus producing one-dimensional models. An interesting special case of such a 1-D model is the one described by the equation:

***output*(*k*)^(*t*) ^**= ***output*(*k*)^(*t*-1) ^*δ*(*S*_2_) ⊕ *δ*(*S*_2 _- 1),**

where ***S*_2 _**is the sum over 2 neighbor cells of the ***k***-th cell (from the left and from the right).

**Figure 8:**
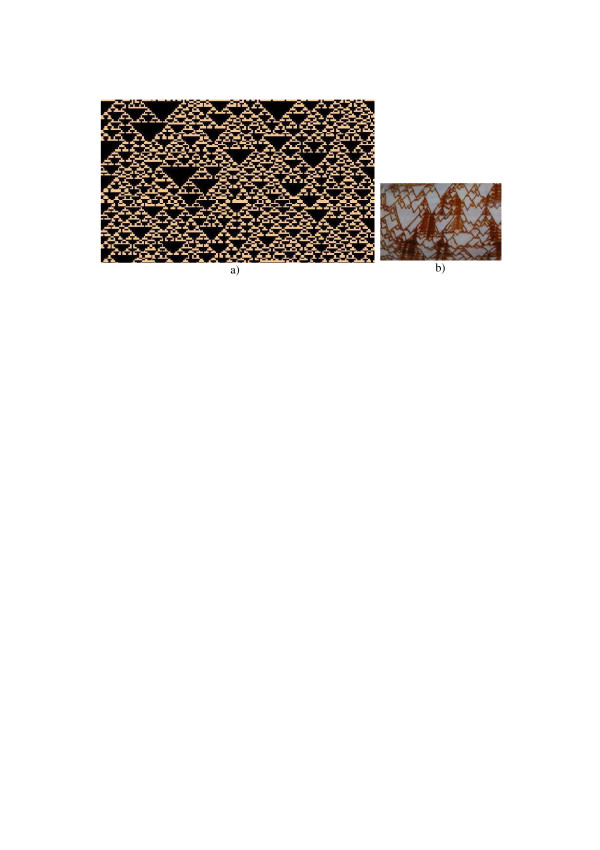
a) – Evolution (downward in vertical direction) of a one-dimensional (in horizontal direction) modification (Eq. 5) of the Game of Life. Initial rate of "alive" points in the first row is 0.3. b) – a fragment of "liva porphiria" see shell.

Figure 8a) shows, row by row, an example of the evolutionary behavior of such a one-dimensional model. It is interesting to observe that patterns, which appear in the process of the evolution, are identical to the so-called Sierpinski Gasket [16]. As it is shown in Figure 8b), they also remind patterns that some see-shells develop in their life (see, for instance, [17,18].

One can further modify the canonical Conway's model by introducing stochastic "death" and "birth" events:

output(k,l)t=2Drandb(Pd)[output(k,l)t−1]δ(S8t−1−2)+2Drandb(Pb)δ(S8t−1−3),
 MathType@MTEF@5@5@+=feaafiart1ev1aaatCvAUfKttLearuWrP9MDH5MBPbIqV92AaeXatLxBI9gBaebbnrfifHhDYfgasaacH8akY=wiFfYdH8Gipec8Eeeu0xXdbba9frFj0=OqFfea0dXdd9vqai=hGuQ8kuc9pgc9s8qqaq=dirpe0xb9q8qiLsFr0=vr0=vr0dc8meaabaqaciaacaGaaeqabaqabeGadaaakeaaieWacqWFVbWBcqWF1bqDcqWF0baDcqWFWbaCcqWF1bqDcqWF0baDdaqadaqaaiab=TgaRjabcYcaSiab=XgaSbGaayjkaiaawMcaamaaCaaaleqabaGae8hDaqhaaOGaeyypa0dcbeGae4NmaiJae4hiaaIae4hraqKae4NCaiNae4xyaeMae4NBa4Mae4hzaqMae4Nyai2aaeWaaeaacqWFqbaudaWgaaWcbaGae8hzaqgabeaaaOGaayjkaiaawMcaamaadmaabaGae83Ba8Mae8xDauNae8hDaqNae8hCaaNae8xDauNae8hDaq3aaeWaaeaacqWFRbWAcqGGSaalcqWFSbaBaiaawIcacaGLPaaadaahaaWcbeqaaiab=rha0jabgkHiTiab+fdaXaaaaOGaay5waiaaw2faaGGadiab9r7aKnaabmaabaGae83uam1aa0baaSqaaiab+Hda4aqaaiab=rha0jabgkHiTiab+fdaXaaakiabgkHiTiab+jdaYaGaayjkaiaawMcaaiabgUcaRiab+jdaYiab+bcaGiab+reaejab+jhaYjab+fgaHjab+5gaUjab+rgaKjab+jgaInaabmaabaGae8huaa1aaSbaaSqaaiab=jgaIbqabaaakiaawIcacaGLPaaacqqF0oazdaqadaqaaiab=nfatnaaDaaaleaacqGF4aaoaeaacqWF0baDcqGHsislcqGFXaqmaaGccqGHsislcqGFZaWmaiaawIcacaGLPaaacqGGSaalaaa@821E@

where **2Drandb(*P*_*d*_) **and **2Drandb(*P*_*b*_) **are the same binary pseudo-random number generators as in the Eden's model (Eq. 2). They produce "ones" with probabilities ***P*_*d *_**(probability of "death") and ***P*_*b *_**(probability of "birth"), respectively. Note that in the original, non-stochastic, Conway's model, ***P*_*d *_**= ***P*_*b *_**= **1**.

**Figure 9:**
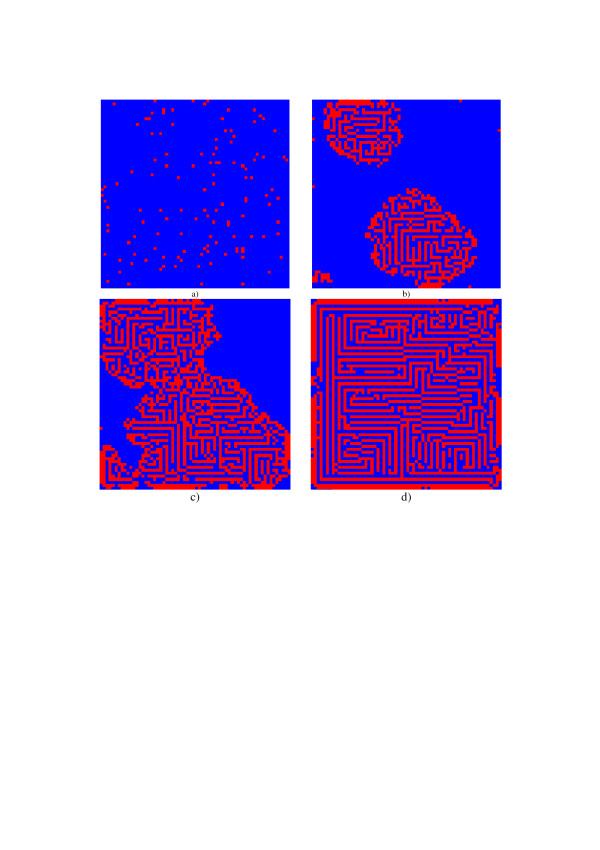
Examples of the modified Conway's model evolution with ***P***_*d *_= **0.25 **and ***P*_*b *_**= **1 **(a) – initial pattern; b), c), d) – evolution results after 50, 75 and 200-th iteration steps, correspondingly, that form "labyrinth" or "zebra skin" – patterns.

**Figure 10:**
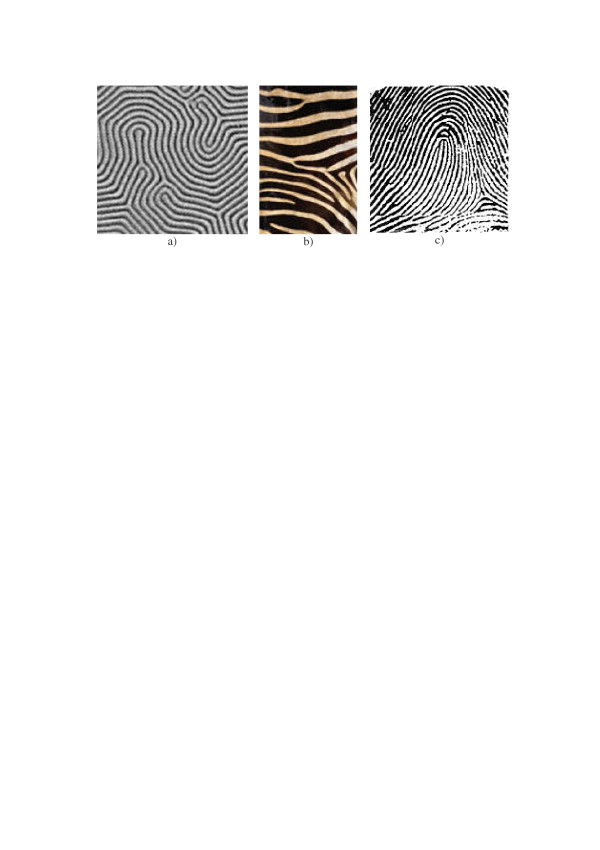
Natural "labyrinth" and "zebra skin" patterns. a) – image of the magnetic domain structure (adopted from [21]); b) – a fragment of the zebra skin; c) – a fingerprint.

If ***P*_*b *_**<**1 **the evolutionary behavior of this model changes very substantially. The model begins to produce labyrinth-alike formations with irregular dislocation whose positions depend on the realization of the initial primary pattern. While the "body" of the patterns stabilizes after a few iterations, their periphery continues growing independently until the pattern fills the entire lattice. Depending of the probability of "ones" in the initial pattern it may happen that several such formations arise and start growing until they merge into one larger labyrinth-alike formation. An example of such an evolution is shown in Figure 9. These labyrinth-alike patterns can very frequently be found among natural patterns such as patters of magnetic domains, paterns of stripes on zebra skin, labyrinth alike patterns on fingerprints and similar formations (Figure 10 adopted from [21]).

One can further generalize the Conway's model in different ways. An interesting option is the one, in which the Kronecker delta-function ***δ*(·)**, which describes logical operation in Eq. 4, is replaced by a "fuzzy delta", a non-monotonic unimodal function **Δ(·) **[9-11]:

output(t)(k,l)=[output(t−1)(k,l)]Δ(L1(t−1)−C1)+Δ(L2(t−1)−C2)
 MathType@MTEF@5@5@+=feaafiart1ev1aaatCvAUfKttLearuWrP9MDH5MBPbIqV92AaeXatLxBI9gBaebbnrfifHhDYfgasaacH8akY=wiFfYdH8Gipec8Eeeu0xXdbba9frFj0=OqFfea0dXdd9vqai=hGuQ8kuc9pgc9s8qqaq=dirpe0xb9q8qiLsFr0=vr0=vr0dc8meaabaqaciaacaGaaeqabaqabeGadaaakeaaieWacqWFVbWBcqWF1bqDcqWF0baDcqWFWbaCcqWF1bqDcqWF0baDdaahaaWcbeqaamaabmaabaGae8hDaqhacaGLOaGaayzkaaaaaOWaaeWaaeaacqWFRbWAcqGGSaalcqWFSbaBaiaawIcacaGLPaaacqGH9aqpdaWadaqaaiab=9gaVjab=vha1jab=rha0jab=bhaWjab=vha1jab=rha0naaCaaaleqabaWaaeWaaeaacqWF0baDcqGHsislieqacqGFXaqmaiaawIcacaGLPaaaaaGcdaqadaqaaiab=TgaRjabcYcaSiab=XgaSbGaayjkaiaawMcaaaGaay5waiaaw2faaGGabiab9r5aenaabmaabaGae8htaW0aa0baaSqaaiab+fdaXaqaamaabmaabaGae8hDaqNaeyOeI0Iae4xmaedacaGLOaGaayzkaaaaaOGaeyOeI0Iae83qam0aaSbaaSqaaiab+fdaXaqabaaakiaawIcacaGLPaaacqGHRaWkcqqFuoardaqadaqaaiab=XeamnaaDaaaleaacqGFYaGmaeaadaqadaqaaiab=rha0jabgkHiTiab+fdaXaGaayjkaiaawMcaaaaakiabgkHiTiab=neadnaaBaaaleaacqGFYaGmaeqaaaGccaGLOaGaayzkaaaaaa@6E16@

where ***L*_1 _**and ***L*_2 _**are outputs of linear filters that replace summations over 8 neighbors in the model of Eq. 4 and ***C*_1 _**and ***C*_2 _**are constants that replace thresholds 2 and 3 in the model of Eq. 4. In this modification, states of cells are not binary and are modeled by real numbers that take arbitrary values in the range [0,1].

Experiments reveal very rich evolutional pattern formation capability of this model. With this model, the following three major types of the evolutionary behavior can be observed depending on the spread of the "fuzzy delta" and constants ***C*_1 _**and ***C*_2_**: "stable chaos", "ordering of chaos" and "reemerging of chaos".

In the "stable chaos" mode, initial chaotic patterns produced by the primary 2-D random number generator gradually evolve into visually correlated patterns that then remain to look similarly though individual cell values keep changing with iterations.

In the "ordering of chaos" mode, initial chaotic patterns degenerate, in the course of iterations, into spatial star constellation-alike or labyrinth-alike patterns that remain stable spatial-wise but may exhibit "temporal" (iteration-wise) cycles. Obviously these are the model's "fixed" points.

The most complex and varying is the behavior of the "reemerging of chaos" type. Its basic feature is rapid degeneration of the initial pseudo-random pattern into a uniform field (a trivial fixed point of the model) or into "star constellations". After that, a new chaotic pattern emerges through growing crystal-alike formations from the constellations left from the initial pattern, through spatial waves from the borders when they are kept to be random, or through the appearance of different types of "gliders" that move across and collide producing clouds of new "particles". These emerging formations gradually fill in the field with visually correlated patterns similarly looking to those, which are characteristic for the "stable chaos" mode.

**Figure 11:**
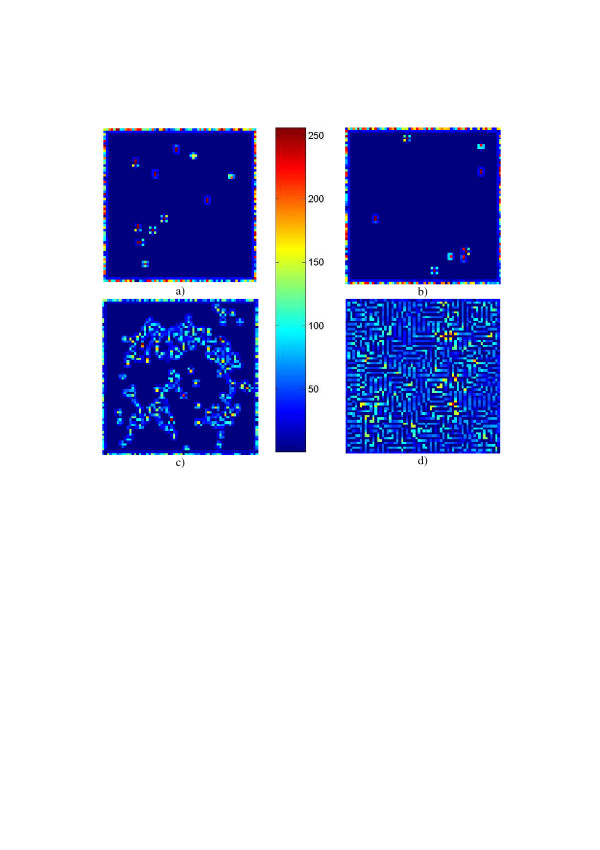
Examples of the evolutionary behavior of the modified Conway's model of Eq. 7: stable "star constellations" patterns (a,b), "clouds" (c), and labyrinth-alike pattern (). Cell value levels in the images are varying here from 0 to 255 and are coded in color as it is represented by the color bar.

**Figure 12:**
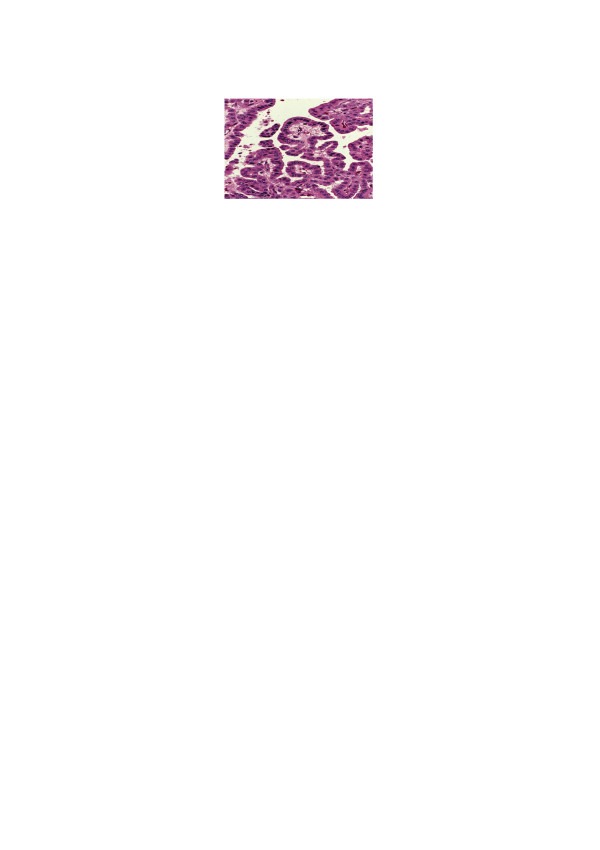
Natural "oncocytic papillary pattern“ (adopted from [23]).

Examples of the evolutionary behavior of such a model are shown in Figure 11. As one can see, typical examples of patterns generated by the model are labyrimth patterns (Figure 11d) and papillary pattern (Figure 11c) that remind patterns frequently found in cytology as the one shown in Figure 12 (adopted from [23]). An illustrative video showing moving gliders generated by the model can be found at L. Yaroslavsky’s home page ([22]).

## 2-D pattern formation: texture images

**Figure 13:**
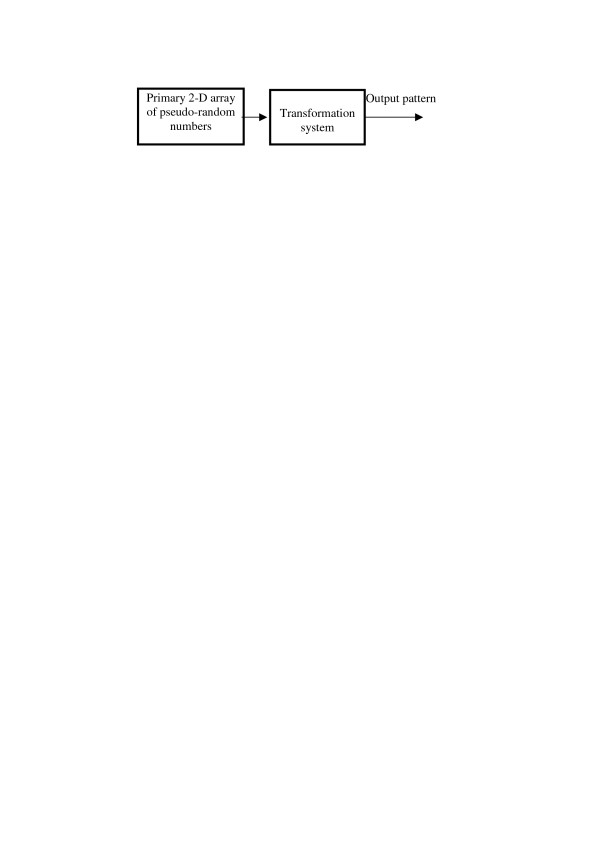
Algorithmic model of texture images.

Above described growth models can be regarded as special cases of a general model described by schematic diagram shown in Figure 13. Such a representation assumes that output patterns are generated by mean of a transformation of primary 2D arrays of pseudo-random numbers in a certain signal processing system. Different systems produce patterns of different classes. Patterns generated by the same system out of different realizations of the primary array of pseudo-random numbers are different realizations of patterns of the same class defined by the transformation system.

In order to make such a representation constructive, we will assume that the transformation systems are built from of a set of certain standard (elementary) signal processing units. Parameters of these units and the transformation system structure form the set of parameters that define stochastic pattern of a certain class. Specific selection of the set of structural signal processing units is governed by considerations of the convenience of their parameterization and by their computational complexity. It is only natural to use units that form the basic and computationally efficient instrumentation tool of digital signal and image processing (see, for instance, [19], such as following units:

• Point-wise nonlinearity (PWN) that transforms signal samples according to the relationship:

***output*(*k*, *l*) **= ***F*(*input *(*k*, *l*)),**

where ***F*(·) **is, generally, a nonlinear function that defines the transformation transfer function of the unit and **(*k*, *l*) **are sample indices.

• Linear filters (LF). Linear filters are defined by the equation of weighted summation:

output(k,l)=∑m,nh(m,n;k,l)input(k,l),
 MathType@MTEF@5@5@+=feaafiart1ev1aaatCvAUfKttLearuWrP9MDH5MBPbIqV92AaeXatLxBI9gBaebbnrfifHhDYfgasaacH8akY=wiFfYdH8Gipec8Eeeu0xXdbba9frFj0=OqFfea0dXdd9vqai=hGuQ8kuc9pgc9s8qqaq=dirpe0xb9q8qiLsFr0=vr0=vr0dc8meaabaqaciaacaGaaeqabaqabeGadaaakeaaieWacqWFVbWBcqWF1bqDcqWF0baDcqWFWbaCcqWF1bqDcqWF0baDdaqadaqaaiab=TgaRjabcYcaSiab=XgaSbGaayjkaiaawMcaaiabg2da9maaqafabaGae8hAaG2aaeWaaeaacqWFTbqBcqGGSaalcqWFUbGBcqGG7aWocqWFRbWAcqGGSaalcqWFSbaBaiaawIcacaGLPaaacqWFPbqAcqWFUbGBcqWFWbaCcqWF1bqDcqWF0baDdaqadaqaaiab=TgaRjabcYcaSiab=XgaSbGaayjkaiaawMcaaaWcbaGae8xBa0MaeiilaWIae8NBa4gabeqdcqGHris5aOGaeiilaWcaaa@593A@

where ***h*(*m*, *n*; *k*, *l*) **– is the filter impulse response.

• Rank filters (RF) [19]. Rank filters operate with signal order statistics computed over a certain neighborhood of each sample of the array and are defined by the equation:

***output*(*k*, *l*) **= **F_*los*_(*input*(*k*, *l*)),**

where **F_*los*_(·) **is a function defined by the local order statistics computed, for every (***k*, *l***)-th sample of the array over its certain neighborhood (***nbh***).

• Logical filters. Logical filters assume working with binary arrays and are defined by a certain Boolean function of input pixels. For binary images, logical filters can implement both linear and rank filters.

This list of signal processing elementary units does not pretend to be complete and one is free to further extend or to modify this list to include other processing units proved to be useful signal processing components. As for the connection of units into a system, the following types of interconnections can be assumed:

• serial connection;

• parallel connection;

• feedback.

In what follows in this section, we will show how such an approach allows to readily build models that are capable of generating very wide variety of stochastic 2-D patterns, including those that imitate natural textures. We will call these patterns texture images.

**Figure 14:**
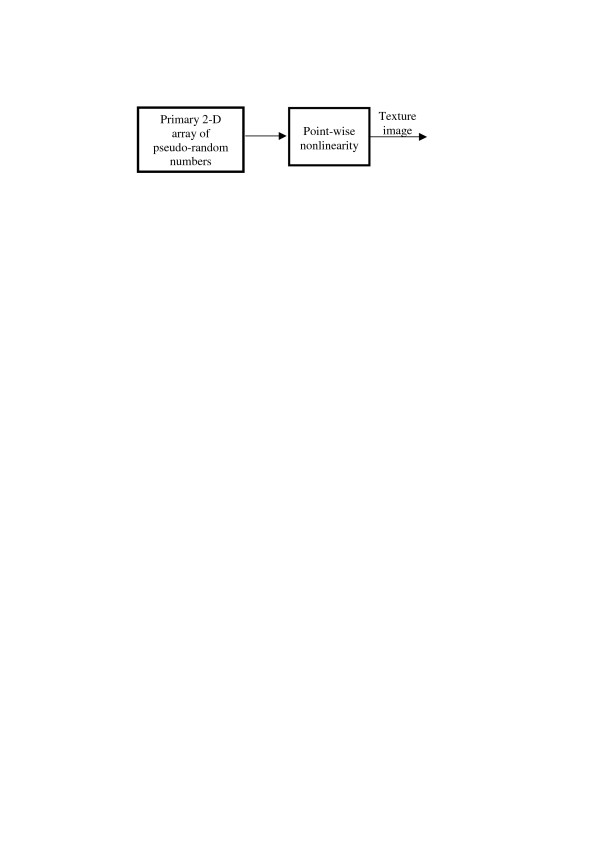
PWN-model of texture images.

Figure 14 represents the simplest PWN-model, in which the transformation system consists of the primary pseudo-random random generator and a single point-wise nonlinear transformation (PWN) unit in cascade. Note that unit **2Drandb(*P*) **used in the above described growth models is a particular version of this model, in which point-wise nonlinearity is a threshold nonlinearity

In the PWN-model, one can easily, by an appropriate selection of the nonlinearity, control probability distribution density of the values of samples of generated patterns.

**Figure 15:**
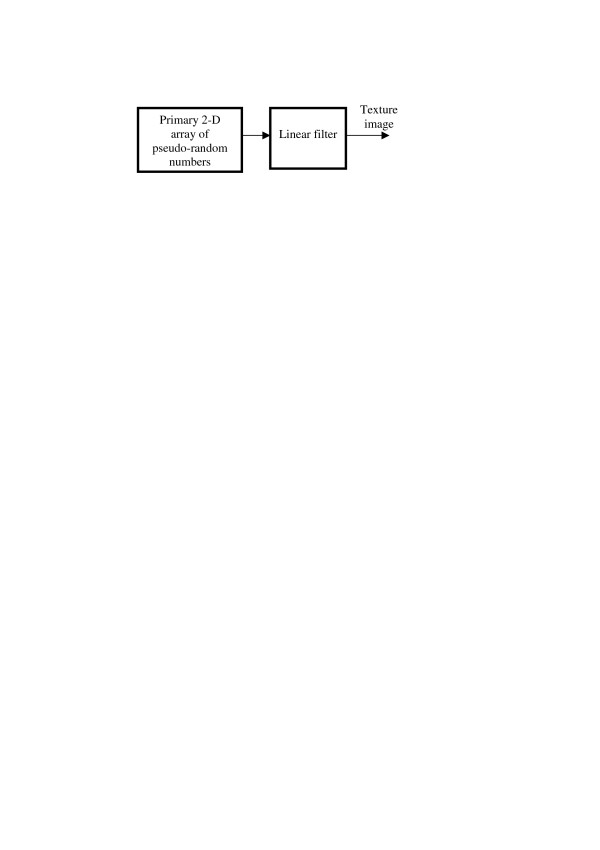
LF-model of texture images.

The next step in the hierarchy of models is LF-model, in which the transformation system consists of a primary pseudo-random number generator and a linear filter in cascade (Figure 15).

On can show [11,19] that, with LF models, patterns with probability distribution of sample values close to the Gaussian distribution are generated. Selection of the linear filter frequency response (Fourier Transform of its impulse response) controls Fourier power spectrum (spectral density) of the pattern and, correspondingly, its correlation function.

**Figure 16:**
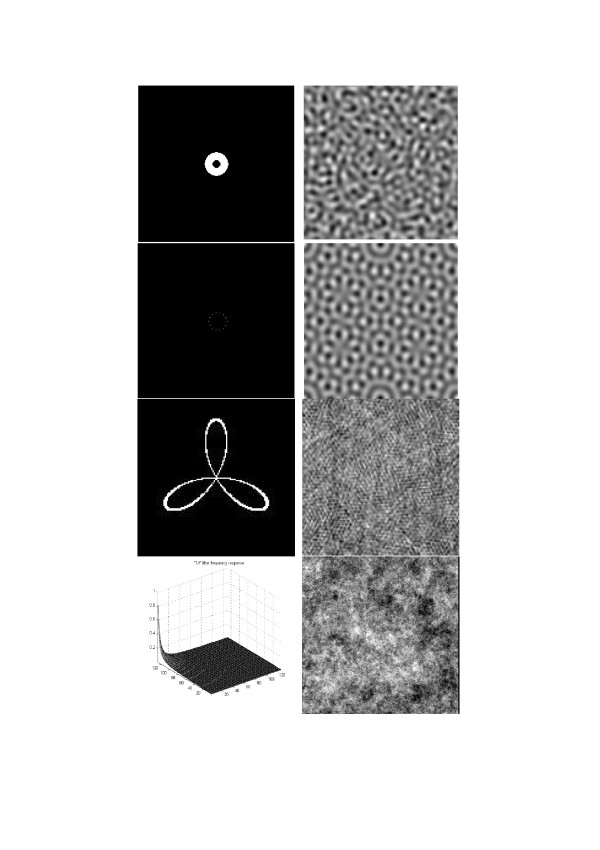
Examples of texture images generated by LF-models (right column) and the corresponding filter frequency responses shown (left column) in form of images and as a plot in 2-D coordinates of spatial frequencies.

Figure 16 shows four examples of patterns obtained from initial pattern of uniformly distributed uncorrelated pseudo-random numbers using linear filters with different frequency responses (shown in the left column of the figure). Especial interest represents the texture shown at the bottom of the figure. It was generated using linear filter with the isotropic filter frequency response, inversely proportional to absolute value of spatial frequency. This texture image illustrates what is conventionally known as **(*1/f*)**-fractals [16].

**Figure 17:**
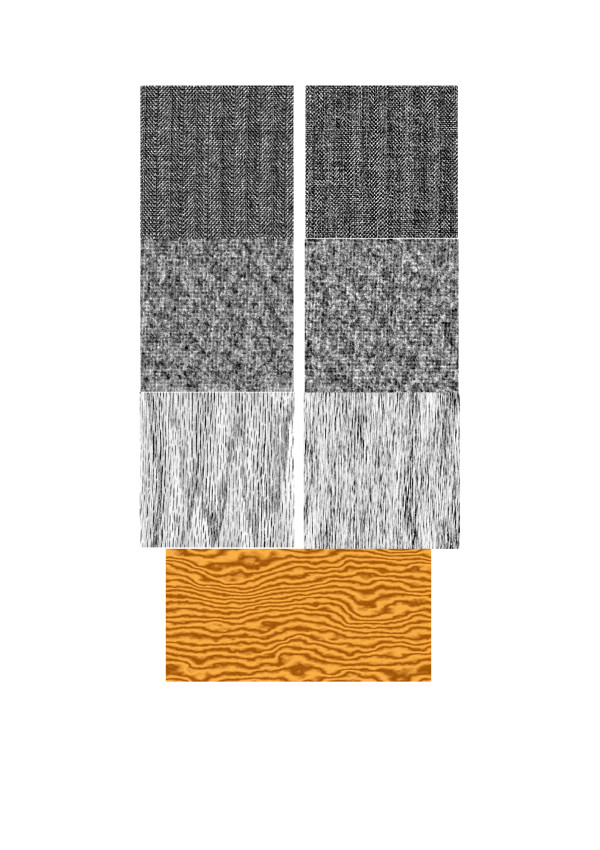
Natural texture images from Brodatz' album of natural textures [24] (left column, from top to bottom: textile, moher, wood) and their synthetic copies (right column) generated by the LF-model. Image at the bottom is yet another example of a synthetic wood-texture artificially colored in brown.

LF-model, however simple it is, allows imitating quite a number of natural texture images [11,20]. Some illustrative examples of such images are shown in Figure 17, [24].

**Figure 18:**
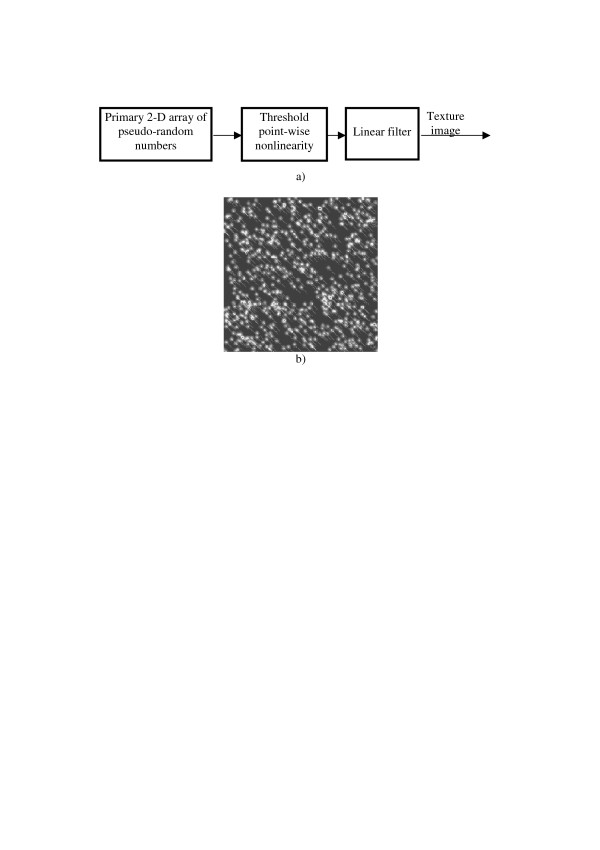
PWN-LF-model of texture images (a) and an example of a generated texture (b).

Combination of the threshold type point-wise nonlinearity and a linear filter in cascade with the primary pseudo-random number generator (Figure 18a) forms PWN-LF models. They generate patterns of randomly distributed filter impulse responses. An example is shown in Figure 18b).

**Figure 19:**
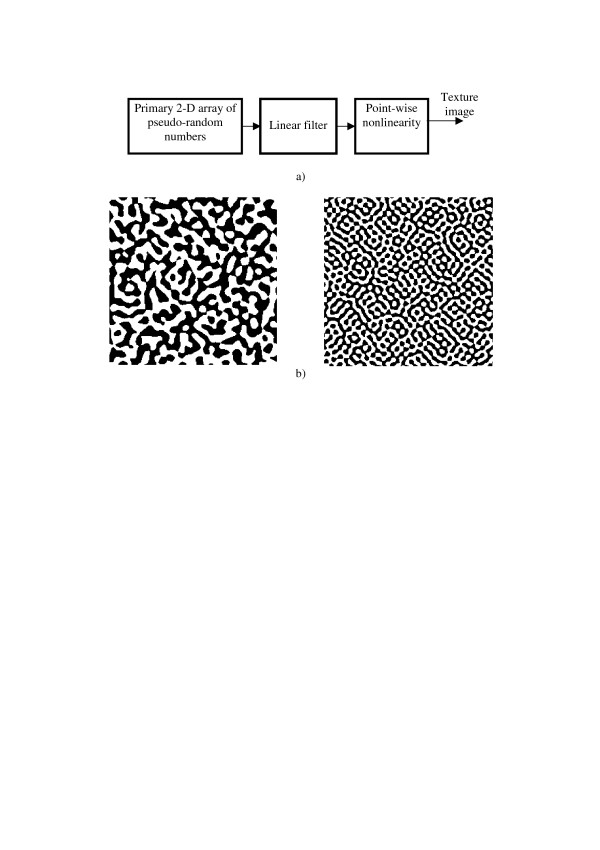
LF-PWN-model (a) and examples of generated texture images (b).

Inversion of the order of the point-wise nonlinearity and linear filter in PWN-LF models results in LF-PWN-models (Figure 19a). LF-PWN-models allow to generate textures with correlation function controlled by the linear filter impulse response and with a given distribution density controlled by the nonlinear unit. Examples of such a texture image is shown in Figure 19b).

**Figure 20:**
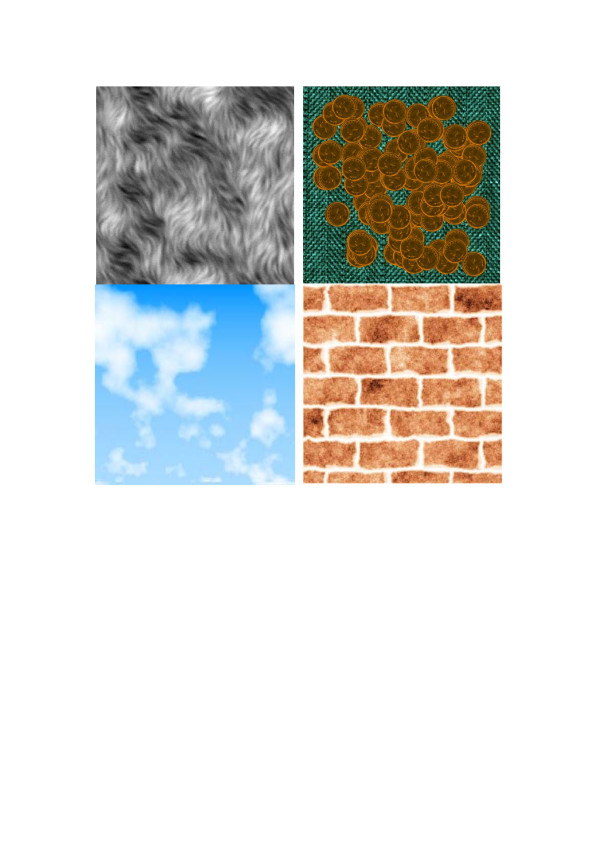
Naturally looking texture images generated by models with multiple branches.

All above described models contain only one branch (several units in cascade). Obviously, texture models can have several branches whose outputs can be combined in different ways. For instance, outputs of branches can be multiplied, or output of one branch can be used to switch between outputs of other branches, or output of one branch can control parameters of the transformation units in another branch, etc. Above described growth models of Figure 3 and 4 exemplify such multiple branch models. Figure 20 shows four examples of texture images generated by more sophisticated models with multiple branches.

Up to now no feedback connection was assumed in the texture models except the one in the primary pseudo-random number generator. Clearly, feedback gives to the models evolutionary features In order to exhibit nontrivial evolutionary behavior, systems should contain, in a loop, both linear filter and a non-monotonic nonlinearity or a nonlinear filter with spatial interaction, such as rank filter. Inserting into the loop rank filters that combine spatial interaction and substantial nonlinearity in a more sophisticated way then just by cascading linear filters and point-wise nonlinearity gives rise to a new family of evolutionary models. Note that the primary pseudo-random number generator in such system serves to introduce only an initial "seed" pattern. The above random number generator of Figure 2 and growth models exemplify the simplest of such systems.

**Figure 21:**
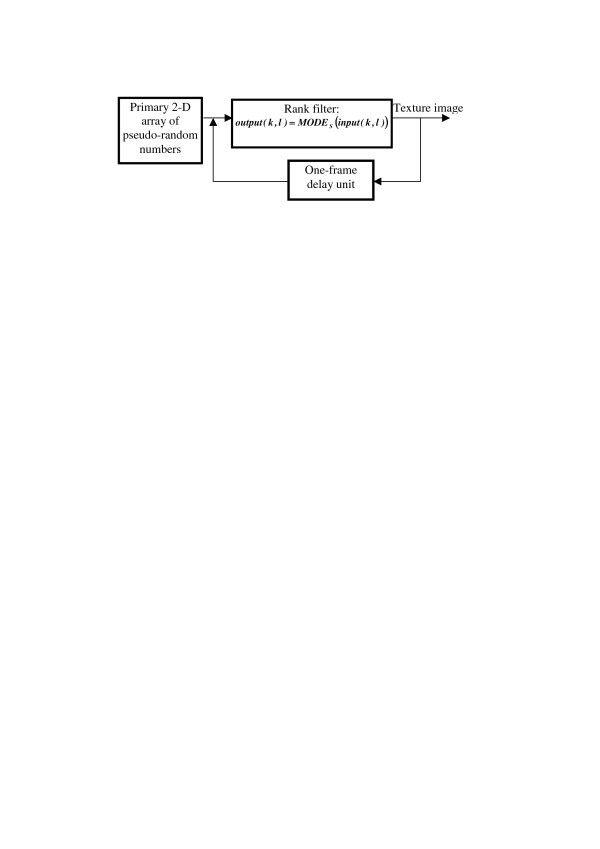
Schematic diagram of an evolutionary texture model with a rank filter MODES.

**Figure 22:**
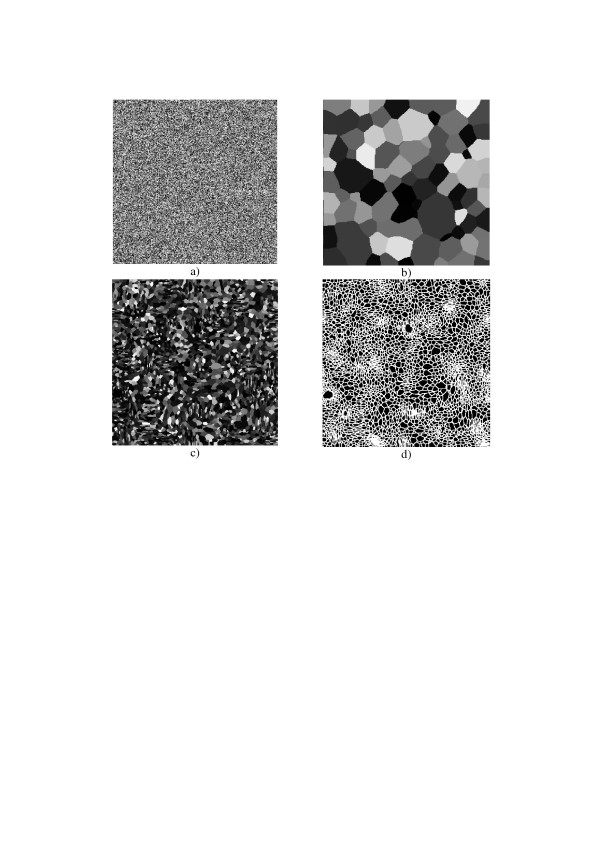
Examples of images generated by the model of Figure 20: primary pseudo-random pattern (a); two texture images generated by the special homogeneous (b) and inhomogeneous (c) model with different size of the spatial neighborhood; edges pattern of figure (d).

**Figure 23:**
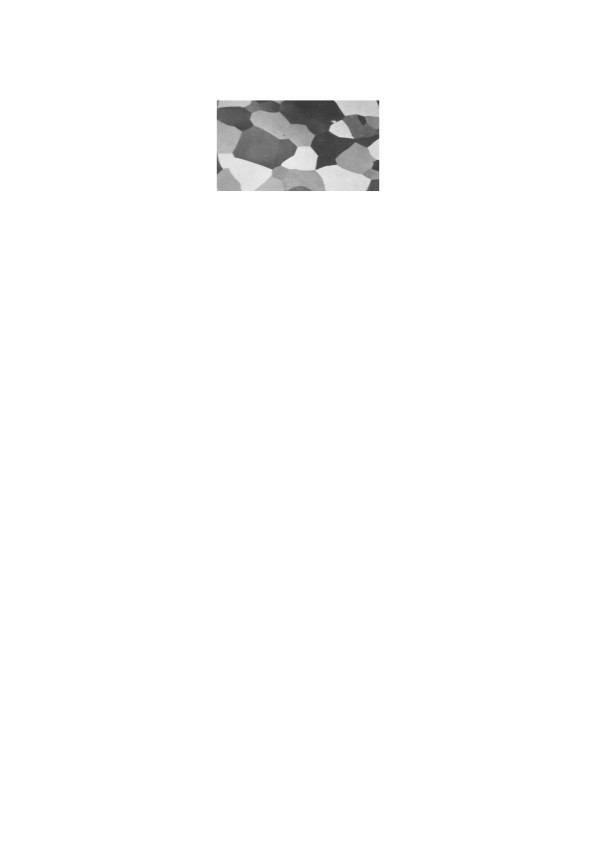
Natural texture image of crystals.

An example of such an evolutionary model with a rank filter is illustrated in Figs. 21. Figure 22 shows examples of textures generated by the model. They very closely remind natural patterns of crystals illustrated in Figure 23. The rank filter used in this model replaces, in each iteration, gray level of every pixel by the most frequent value within a certain spatial window ***S ***centered at the pixel (this operation is called ***MODE*_S _(*input*(*k*, *l*) **with ***k*, *l ***as pixel 2-D indices[19]).

As one can see, patterns generated by this model are very reminiscent of natural crystals, cells and cell wall patterns.

## Conclusion

We outlined an approach to the analysis and design of stochastic growth and pattern formation models that treats the models in terms of nonlinear signal processing systems with feedback composed of a set of standard and algorithmically simple processing units. We have described a variety of concrete growth and pattern formation models built on the base of this approach and have shown, on examples, that they are capable of imitating natural growth and patterns such as dendrites, see shell, labyrinth, zebra skin, papillary, fingerprint patterns, fur, wood, textile, clouds and alike textures. We believe that such a unified approach facilitates the growth and pattern formation model design, comparison, quantification and unification and secures their efficient computational implementation.
